# Quality of life in men with metastatic prostate cancer in their final years before death – a retrospective analysis of prospective data

**DOI:** 10.1186/s12904-018-0381-6

**Published:** 2018-12-03

**Authors:** Maja Holm, Sandra Doveson, Olav Lindqvist, Agneta Wennman-Larsen, Per Fransson

**Affiliations:** 1 Department of Nursing Sciences, Sophiahemmet University, Box 5606, 114 28 Stockholm, Sweden; 2 Division of Insurance Medicine, Department of Clinical Neuroscience, Karolinska Institutet, 171 77 Stockholm, Sweden; 3 Department of Learning, Informatics, Management & Ethics, Karolinska Institutet., 171 77 Stockholm, Sweden; 4 Department of Nursing, Umeå University, 901 87 Umeå, Sweden; 5 Cancercentrum, Norrlands University Hospital, 901 85 Umeå, Sweden

**Keywords:** Prostate cancer, Metastatic disease, Palliative care, Quality of life, Life-prolonging treatment

## Abstract

**Background:**

Quality of Life (QoL) is the most important outcome for patients in palliative care along with symptom alleviation. Metastatic prostate cancer (mPC) is a life-threatening illness, and hence, a palliative care approach may be beneficial to this group. Over time, new life-prolonging treatments have been developed for men with mPC, but the possibility to prolong life should also be balanced against the men’s QoL, particularly because there are side effects involved with these treatments. The aim of this study was to evaluate QoL, functioning and symptoms in men with mPC during their final years before death.

**Methods:**

This is a retrospective analysis of data from a long-term prospective study of men (*n* = 3885) with prostate cancer from two regions in Sweden. Validated questionnaires asking about participants’ QoL, functioning and symptoms were used to collect data. From the overall study, 190 men with mPC were identified. They were stratified into three groups, depending on the amount of time that had passed between the last questionnaire and their death; < 6 months, 6–18 months and > 18 months before death.

**Results:**

Men with mPC generally rated their QoL poorly compared to established clinically significant threshold values. The group of men that were < 6 months before death rated their QoL, functioning and several symptoms significantly worse than the two other groups. Men that died after the year 2006 reported lower QoL and functioning and more pain and fatigue than those who died before 2006.

**Conclusion:**

The results in this study indicate that men with mPC have unmet needs with regards to QoL and symptoms. A palliative care approach, alongside possible life-prolonging treatments, that focuses on QoL and symptom relief, may serve as an important frame to give the best support to these men in their final years of life.

## Background.

Prostate cancer (PC) is a common cancer type in men in the OECD-countries that take many lives yearly [1, 2]. In Sweden, 10,439 men were diagnosed with PC and 2357 men died because of the disease in 2015 [3].

Most men with PC are diagnosed with localized disease. However, 10–20% of the men are diagnosed with metastatic disease (mPC) and 20–30% will develop metastases during the course of illness [4]. It is most common that PC metastasizes to bone (particularly the pelvis and spine), which can lead to incapacitating pain and fractures. Bone metastases occur in 70% of men with advanced disease and are present in 90% of men who die from PC. Other symptoms associated with mPC are fatigue and problems with urinary- and sexual functioning [5]. Over the last decade, the treatment of men with mPC has progressed, with new therapies contributing to increased survival even in men that have become resistant to hormone treatment [6]. Symptoms such as diarrhoea and nausea/vomiting are known side-effects associated with these life-prolonging treatments [7]. Hence, men with mPC may require a palliative care approach focused on promoting quality of life (QoL) and symptom relief [8] early in the course of illness and alongside life-prolonging treatments [9].

Although there are many studies on QoL and symptoms in men with PC, most of them are from the early stages of the disease and often related to curative treatment, or from a palliative care context in the final weeks of life. Very few studies have focused on men with advanced PC earlier during their illness trajectory. A mini-review concluded that research should place more focus on patient-centred outcomes and not just on patient survival [10]. A study from 2008 [11] demonstrated that men with mPC had various forms of pain, fatigue and sexual problems but that they were not necessarily distressed by their symptoms. Qualitative studies have concluded that advanced PC affects men’s lives: they are placed in a new life situation, against their will, with increasing bodily changes and symptoms and in their new situation they form a new life perspective [12, 13].

PC has been described as particularly suited to palliative care, as the progress of the disease is often long and slow compared to other forms of cancer [8]. A Cochrane review showed that an early integration of palliative care principles can improve QoL and reduce the symptom burden in patients with advanced cancer. These principles can also be applied in combination with life-prolonging treatments, such as chemotherapy or radiation therapy [14]. In Sweden, palliative care has been given increased priority over the last decade. In 2006, a report was presented by the National Board of Health and Welfare focusing on the development of palliative care in Sweden and the needs for improvement [15]. In the same year, a national quality register for palliative care was also introduced [16]. Since then, palliative care services in Sweden have expanded greatly. In a study from 2016, it was found that palliative care services in Sweden had been significantly developed between 2005 and 2012 and that the rate of palliative home care teams had increased from 0.55 to 1.13 per 100,000 citizens, making the coverage one of the highest in Europe [17].

Even if the life-prolonging treatment options for men with mPC have changed dramatically in recent years, from a palliative care perspective, the prolonged life-expectancy must be balanced against men’s QoL in this advanced stage of the disease. Therefore, the relevance of evaluating palliative care outcomes, like QoL, pain and fatigue, as well as physical- and emotional functioning are of essential importance. Further, more prostate specific outcomes like urinary and bowel problems and sexual functioning should also be assessed in this group [18]. Conclusively, there is a need for more knowledge about men with life-threatening mPC and their life situation. Hence, the aim of this study was to evaluate QoL, functioning and symptoms in men with mPC in their final years before death.

## Methods

### Data collection

Data were taken from a prospective study [19] of 3885 men with primary localized PC from two regions in Sweden. Data were collected through repeated questionnaires and from medical records. Inclusion criteria for the prospective study were; being diagnosed with localized PC and scheduled for treatment, with radiotherapy, with or without hormonal therapy, or radical prostatectomy. The men were included between the years 1992 and 2007. Upon inclusion, they were all listed with a cancer specialist physician at their oncology unit and had access to a social worker for support if needed. They were followed up to twenty years after inclusion until 2017. From the patient’s records, information was collected regarding time of diagnosis, date of treatment and death. Questionnaires were distributed before and after the start of primary treatment and then 3 months and 1, 3, 5, 8, 10, 12, 15 and 20 years after inclusion. The patients were invited to participate in the study by a nurse at the radiotherapy or urology department before their first treatment. Patients were asked to complete the questionnaire and to return it to the clinic staff. In the subsequent follow-ups, patients received the questionnaires by mail and after completion they were returned by mail in prepaid envelopes. A reminding letter was sent to those who did not respond within 4 weeks.

Because the present study had a focus on patients with advanced disease that may have palliative care needs, a subsample was selected, which only included patients with mPC, that had died during their follow up period up until October 2017. The focus of the present study was to retrospectively analyse the patient’s situation before death and hence, only the last questionnaire before the patient’s death was used. To evaluate the situation for patients in various stages of their disease, the patients were stratified into three groups depending on the amount of time that had passed between the final questionnaire and their death; died < 6 months after the last questionnaire; died 6–18 months after the last questionnaire and died > 18 months after the last questionnaire.

### Measurements

The *European Organization of Research and Treatment of Cancer Quality of life Questionnaire version 3.0* (EORTC-QLQ-C30) [20] is an instrument designed to measure Global health/ QoL in patients with cancer. It is a 30-item scale composed of five functional scales, three symptom scales, a global health status and six single item symptom measures. There are four response alternatives (“not at all”, “a little”, “quite a bit” and “very much”), except for global health/QoL, that has seven response options, ranging from “very poor” to “excellent”. Scores are calculated either by scale or by item and transformed into a 0–100 scale. High values indicate either good functioning or high symptomatology [21]. The instrument has been validated for patients with cancer [22]. For the present study, items/scales measuring QoL, social, cognitive, emotional, physical and role functioning, fatigue, pain, nausea, dyspnoea, insomnia, constipation, loss of appetite and diarrhoea were used. In this study, the men’s scores were compared against threshold values that have been established for some of the scales in previous studies. These values indicate the need for attention from clinicians [23–25].

Three items from *The Prostate Cancer Symptom Scale (PCSS)* [26] were also used in this study*.* This questionnaire was developed to measure the level of distress by PC specific symptoms and includes 56 items distributed over four categories (general symptoms, bladder-, bowel symptoms, and sexual function). The instrument uses a modified linear analogue scale ranging between 0 and 10, where 0  =  “no problem/very good function” and 10  =  “many problems/very bad function”. The three questions that were used for this study were: *“Do you have any urinary problems?” “Do you have any bowel problems?”* and *“Do you have any problems with your sex life?”*

### Data analysis

Statistical analyses were conducted using Stata 13.1 (StataCorp, College Station, TX, USA). For all analyses, *p* ≤ 0.05 was taken to be statistically significant. All statistical tests were two-sided.

Data were initially analysed through descriptive statistics to explore the background characteristics of the three groups of men with mPC in their final years before death. Characteristics included their mean age, a classification of their primary tumour, the proportion receiving hormone treatment and the number of years with PC and mPC. To compare the level of self-rated QoL, functioning and symptoms between the three groups, one-way ANOVA was performed. To identify where the differences lay between the groups, a post-hoc test was performed with Tukey’s method, where the groups were compared pairwise regarding their ratings of the studied outcomes.

Further, the Bonferroni test for multiple comparisons was performed, as several hypotheses were tested, increasing the risk of committing Type-I errors. Since the number of men in the various phases differed and because the variables were somewhat unevenly distributed, it was also decided to run a nonparametric Kruskal Wallis rank sum-test to confirm the results. The results from this analysis was however consistent with the results from the ANOVA and hence the results from the ANOVA are presented in the results section and in Table 2.

Because data was collected over a long period of time, and the options for treatment of PC in advanced stages, as well as the organisation and coverage of specialised palliative care have changed dramatically during these years, it was decided to also compare the outcomes during two time-periods, before 2006 and during or after 2006. This was the median year of death of the sample in this study and was considered a relevant time-point for two reasons. One was that the first life-prolonging treatments for metastatic castration refractory prostate cancer was approved in 2004 [27], but Swedish policy documents indicate that it took some time before these were fully implemented as the first treatment-option for this group of patients [28]. The other was that a national quality register for palliative care was introduced this year, potentially giving focus on these questions from a healthcare perspective [15, 16]. The mean outcomes were compared with t-tests between the group that had died before 2006 and those who had died during or after 2006.

## Results

### Sample characteristics

From the overall sample (*n* = 3885) in the prospective study, 212 (5%) men with mPC were identified. Of them, 13 were registered as still alive in October 2017 and because the focus of this study was on the men’s situation before death, they were excluded from further analysis. Further, 9 men were excluded due to incomplete questionnaires. Hence, the final sample consisted of 190 men. Their mean age was 70.6 years (SD = 7.1) at the time of the last questionnaire. They were stratified into three groups based on the time passing between the last questionnaire and date of death (died < 6 months after the last questionnaire; died 6–18 months after the last questionnaire and died > 18 months after the last questionnaire) (Table 1).

**Table 1:** Characteristics of the study sample of 190 men with metastatic prostate cancer grouped on time from last questionnaire to death

Time between last questionnaire and death		< 6 months		6–18 months		> 18 months		Total	
Frequency (%)		40 (21.0)		64 (33.7)		86 (45.3)		190 (100.0)	
Mean age (SD)		71.5 (7.4)		69.7 (6.3)		71.0 (7.4)		70.6 (7.1)	
Tumour size classification, n (%)									
(Tumour-Nodes-Metastasis system)		20 (50)	20 (50)	41 (64)	23 (36)	53 (62)	33 (38)	114 (60)	76 (40)
1–2	3–4								
Year of diagnosis, Median (q^1^-q^3)^		1998 (1996–2002)		1998 (1994–2001)		1997 (1993–2001)		1998 (1994–2002)	
Year of death, Median (q^1^-q^3)^		2006 (2003–2007)		2005 (2002–2008)		2007 (2003–2011)		2006 (2002–2010)	
Years between PC diagnosis and metastasis, Median (q^1^-q^3)^		5 (2–8)		4 (2.5–6)		5.5 (3–9)		5 (3–8)	
Years between metastasis and death, Median (q^1^-q^3)^		1 (0–2)		2 (1–3)		3 (1–4)		2 (1–4)	
Years with PC, from diagnosis to death, Median (q^1^-q^3)^		6 (3–9)		6.5 (4–10)		9 (7–11)		7 (5–10)	
Proportion (%) receiving hormone treatments upon inclusion		80.0		59.3		62.3		64.6	

### QoL, functioning and symptoms

The average rating of QoL for men with mPC in their final measurement before death was 56.0, which is below the established threshold value for clinical attention of 70. In total, 70.1% of the men in all three groups rated their QoL below the threshold value. However, there were significant differences in the groups’ ratings of their QoL (*p* < 0.001). The post hoc analysis of pairwise means between the groups revealed that the men who died < 6 months after their last questionnaire scored their QoL significantly lower than the other two groups of men (Tables 2 and 3).

**Table 2:** Men with metastatic prostate cancer’s ratings of their QoL, functioning and symptoms (mean, SD, proportions) in relation to clinically relevant threshold values, as well as comparisons between groups based on time from last questionnaire to death *p*-values from One way ANOVA

Variable	Threshold values^d^	Total score			< 6 months before death			6–18 months before death			> 18 months before death			*P*-value^1^
		Mean (SD)	*n*	Prop.^e^ (%)	Mean (SD)	*n*	Prop.^e^ (%)	Mean (SD)	*n*	Prop.^e^(%)	Mean (SD)	*n*	Prop.^e^ (%)	
Global health/QoL^a^	70	56.0 (26.7)	189	70.1	38.5 (27.3)	40	87.5	55.7 (23.5)	64	75.0	64.5 (24.9)	85	59.3	< 0.001
Social functioning^a^		66.8 (29.3)	187		50.4 (32.5)	38		66.4 (27.2)	63		74.4 (26.4)	86		< 0.001
Emotional functioning^a^	70	72.9 (23.4)	188	43.6	59.5 (28.3)	39	67.5	73.6 (19.5)	63	46.9	78.5 (21.4)	86	29.0	< 0.001
Cognitive functioning^a^		78.4 (22.8)	190		67.9 (27.1)	40		78.4 (22.7)	64		83.3 (19.0)	86		0.002
Physical functioning^a^	83	72.9 (28.6)	187	54.5	54.2 (31.8)	38	75.0	71.7 (28.5)	64	56.3	82.2 (22.7)	85	42.4	< 0.001
Role functioning^a^		60.2 (36.7)	173		36.7 (35.0)	34		57.4 (39.4)	61		72.6 (29.5)	78		< 0.001
Fatigue^b^	39	39.2 (29.1)	189	38.6	62.1 (29.7)	39	70.0	39.9 (28.3)	64	40.6	28.2. (22.8)	86	22.1	< 0.001
Pain^b^	25	31.8 (32.1)	190	49.5	53.8 (34.5)	40	77.5	29.4 (29.5)	64	50.0	23.3 (28.3)	86	36.0	< 0.001
Nausea /vomit^b^		10.6 (17.3)	190		23.8 (23.2)	40		8.6 (15.4)	64		6.0 (11.7)	86		< 0.001
Dyspnoea^b^		32.5 (29.5)	189		49.6 (34.9)	39		32.3 (27.2)	64		24.8 (25.1)	86		< 0.001
Insomnia^b^		27.5 (28.7)	189		35.0 (29.2)	40		29.2 (29.4)	64		22.7 (27.3)	85		0.071
Constipation^b^		17.8 (28.7)	187		34.2 (38.1)	40		14.3 (24.5)	63		12.7 (23.7)	84		0.001
Appetite loss^b^		17.9 (27.4)	190		42.5 (32.9)	40		14.6 (24.4)	64		8.9 (18.7)	86		< 0.001
Diarrhoea^b^		15.0 (24.2)	187		18.8 (27.4)	39		10.6 (20.6)	63		16.5 (25.0)	85		0.186
Urinary problems^c^		2.8 (3.1)	184		3.7 (3.2)	38		2.4 (3.1)	63		2.6 (3.0)	83		0.082
Bowel problems^c^		2.7 (2.7)	182		3.7 (2.9)	38		2.8 (2.8)	62		2.2 (2.5)	82		0.027
Sexual problems^c^		6.9 (4.2)	173		7.0 (4.5)	36		7.5 (3.8)	58		6.3 (4.2)	79		0.219

^1^Tested with One-way ANOVA

^a^EORTC-QLQ-C30. Higher values indicate better QoL and functioning

^b^EORTC-QLQ-C30. Higher values indicate more symptoms

^c^PCSS. Higher values indicate more problems

^d^Values that indicate the need for clinical attention

^e^Proportion of values over/under the threshold values

The functional scales ranged between mean 60.2 and 78.4. The mean score for physical function (72.9) was below the threshold value (83) and 54.5% of all men rated their physical function below 83. The < 6 months group rated their social-, emotional, physical and role functioning significantly lower in comparison with the other two groups. The level of cognitive function was also significantly lower in the < 6 months group compared to the > 18 months group (*p* < 0.001), but not in the < 6 months group compared to the 6–18 months group (*p* = 0.08). The difference between the 6–18 months group and the > 18 months was significant for role functioning, but not for any of the other functioning scales.

The symptom scores ranged between 10.6 and 39.2 for all three groups. The mean values for fatigue and pain were above the established threshold values of 39 and 25 respectively, indicating a need for clinical attention. 38.6% of all men in the study rated their fatigue above the threshold value and 49.5% rated their pain over the threshold value. The men who died < 6 months after the last questionnaire reported more symptoms compared to the other two groups. The differences were significant (*p* < 0.05) for all values except insomnia and diarrhoea. The post hoc analysis of pairwise means showed that the < 6 months group had worse scores in all symptom scales except insomnia and diarrhoea than both the 6–18 months and the > 18 months group (Table 3). The comparison between the 6–18 months and the > 18 months group showed that even though the 6–18 months group generally reported more symptoms, the differences were only significant regarding their amount of fatigue (*p* = 0.02).

**Table 3:** Pairwise comparisons in men’s final years before death^a^

Variable	< 6 months, vs 6–18 months *p*-value	< 6 months vs > 18 months *p*-value	6–18 months vs > 18 months *p*-value
Global health/QoL	0.002	< 0.001	0.088
Social functioning	0.017	< 0.001	0.198
Emotional functioning	0.007	< 0.001	0.393
Cognitive functioning	0.052	0.001	0.368
Physical functioning	0.005	< 0.001	0.050
Role functioning	0.016	< 0.001	0.027
Fatigue	< 0.001	< 0.001	0.020
Pain	< 0.001	< 0.001	0.430
Nausea/vomiting	< 0.001	< 0.001	0.590
Dyspnoea	0.008	< 0.001	0.242
Insomnia	0.566	0.066	0.362
Constipation	0.001	< 0.001	0.937
Appetite loss	< 0.001	< 0.001	0.333
Diarrhoea	0.219	0.872	0.309
Urinary problems	0.092	0.120	0.961
Bowel problems	0.325	0.022	0.358
Sexual problems	0.931	0.537	0.216

^a^Tukey posthoc test based on one-way ANOVA

Regarding prostate-specific outcomes, significant differences between the groups were found for bowel problems. The post hoc tests showed that the significant difference was between the < 6 months group and the > 18 months group in their ratings of their bowel problems, where the < 6 months group had more problems (*p* = 0.02) (Tables 2 and 3).

### Comparison between men who died before and after 2006

91 of the men had died before the year 2006 and 99 men had died during or after the year 2006. The men that had died during or after 2006 reported significantly lower ratings of QoL as well as social-, emotional, physical- and role functioning compared to the men that had died before 2006. They also rated their fatigue and their pain significantly higher. There were no significant differences between the two time-periods regarding prostate-specific symptoms (Table 4).

**Table 4:** Comparisons of the outcomes over two periods of time

Variable	Mean (SD) before 2006 (*n* = 91)	Mean (SD) during/after 2006 (*n* = 99)	*P*-value*
Global health/QoL	60.6 (25.3)	51.9 (27.5)	0.030
Social functioning	72.7 (26.2)	61.6 (31.0)	0.009
Emotional functioning	77.5 (21.2)	68.4 (24.4)	0.009
Cognitive functioning	81.7 (19.9)	75.5 (25.0)	0.074
Physical functioning	77.2 (29.0)	68.6 (27.7)	0.047
Role functioning	66.7 (33.1)	55.7 (37.5)	0.048
Fatigue	34.3 (27.6)	44.0 (29.5)	0.029
Pain	26.1 (30.2)	37.2 (33.0)	0.022
Nausea/vomiting	10.0 (18.2)	11.2 (16.5)	0.681
Dyspnoea	30.4 (29.5)	34.0 (29.7)	0.451
Insomnia	21.7 (26.8)	32.6 (29.7)	0.010
Constipation	15.4 (26.0)	20.1 (31.1)	0.300
Appetite loss	14.5 (25.8)	21.1 (28.5)	0.117
Diarrhoea	13.9 (23.9)	16.2 (24.6)	0.492
Urinary problems	2.4 (3.0)	3.2 (3.2)	0.081
Faeces problems	2.6 (2.6)	2.8 (2.9)	0.510
Sexual problems	7.1 (4.1)	6.6 (4.3)	0.308

*T-tests

## Discussion.

The results of this study showed that men with mPC generally rated their QoL and physical functioning poorly, compared to clinically important threshold values. All three groups of men rated their QoL and physical functioning lower than the threshold values, indicating the need for clinical attention. The ratings for pain and fatigue were higher than the threshold values in the groups of men that died < 6 months and 6–18 months before the last questionnaire. Another finding in this study was that men that died during or after 2006 generally rated their QoL and functioning lower and symptoms like pain and fatigue higher than the group that died before 2006.

From a palliative care perspective QoL is the main outcome along with symptom alleviation [29], hence the great proportion of men in all three groups that rated their QoL under the threshold value and their high ratings of symptoms like pain and fatigue compared to threshold values should be taken seriously. In the present study, the men that died < 6 months after the last questionnaire generally rated more symptoms than the two other groups, which of course could be attributed to the more advanced disease. A cancer trajectory usually entails a reasonably predictable decline of the physical health [30] with a more rapid deterioration in the last months before death [31], and in this late phase, increasing bodily symptoms will often be a dominant part of the person’s life [13].

Earlier research has found that a palliative care approach to life-threatening illness could reduce the symptom burden and improve QoL [32], indicating that it may be appropriate for men with mPC [8]. It is also important to stress that even though men who were closer to death had worse ratings, focus should not just be at men with mPC in their final months of life. A Cochrane review found that an early integration of palliative care interventions in advanced cancer could lead to less symptom burden and a higher QoL. Palliative care provides an additional layer of support that can improve QoL also for patients with longer life expectancy and not just supportive care at end of life [5].

No significant differences were found between the groups with regards to urinary or sexual problems, which are typically associated with PC. All men in this sample had been treated with radiation therapy, and since this treatment, as well as castration therapy, have a long-term effect on men’s sexuality and urinary symptoms [33, 34] this may explain that there were no differences between the groups regarding sexual and urinary symptoms.

It is noteworthy that men in the group that died after 2006 rated their QoL and functioning significantly lower and their pain and fatigue higher than the group that died before 2006. After 2006, the palliative care services in Sweden have expanded, focus on palliative care have also increased in other settings, and new policy documents have been presented that give this form of care a high priority [35]. Therefore, it is surprising that men who died after 2006 rate variables that traditionally are given a high focus in palliative care worse than those who died before 2006. A reason behind these ratings could be that new life-prolonging treatments also have side effects [36, 37], which could contribute to lower QoL ratings and higher symptom ratings.

These results highlight the importance of balancing between life expectancy and QoL in treatment decisions for these patients. Symptom relief, particularly for pain, has been declared an essential human right [38], and should be given a high priority in the care of patients with mPC. Screening instruments could be used to capture changes in the patient’s ratings and threshold values could be used to evaluate the need for clinical attention. Although palliative care has traditionally been introduced in late stages of illness, cooperation between oncologists and palliative care teams can take place to provide the best care for men in all phases of mPC. An early integration of palliative care could be offered alongside life-prolonging therapies but also when the patient is approaching the end of life [5, 9]. In conclusion, there is a place for a palliative care approach in all time periods for patients with mPC.

### Strengths and limitations

Strengths of this study included a reasonably large sample of participants and a very long follow-up time. The fact that all data are based on patient reported outcomes is another strength. Both parametric and non-parametric tests were performed, and the nonparametric tests confirmed the results from parametric tests. The results were also confirmed through a correction for multiple comparisons which could reduce the risk of committing Type-1 errors.

There are also limitations to this study, which makes it necessary to be cautious in the interpretation of the results. The EORTC QLQ C-30 was initially developed for studying QoL, function and symptoms as an outcome for anti-cancer treatment in clinical trials and not for patients with advanced disease, although it has been used in several studies in this context. Also, the three groups in this study were not of equal size as the > 18 months group was more than twice as large as the < 6 months group.

The findings are based on just one measurement of men with PC in their last years before death. To further verify the results in this study, it would be interesting to study men with mPC over time, through the various phases before death. Another limitation is the assumption that the men in this study died from mPC. Because information on the actual causes of death was not available in this study, it could be possible that they died for other reasons. It would also have been valuable to have more information on which treatments the men received, as this field has developed rapidly over the last decade.

The division between men who died before and after 2006 was based on the median for this sample and could be considered arbitrary. However, as has been previously stated, this was an important year for the development of palliative care in Sweden. New life-prolonging treatments for mPC had also been presented in 2004 and Swedish policy documents imply that they were not fully implemented until a couple of years later.

## Conclusions

Compared to established threshold values, men with mPC reported poor QoL and functioning particularly in the last few months before death, and a higher level of symptoms like pain and fatigue. The men who died after 2006 had worse QoL, functioning and more pain and fatigue compared to those who died before 2006. Even though many new treatment options have been developed for this group, these results may indicate the challenges in balancing between prolonging life expectancy and striving for best QoL in the last years of life for these men. A palliative care approach, that focuses on QoL and symptom relief, may serve as an important frame to give the best support to these men in their final years of life. A future study interest would be to follow men with mPC over time and through life-prolonging treatments with a focus on QoL, functioning and symptoms.

## Abbreviations

EORTC-QLQ: European Organization of Research and Treatment of Cancer Quality of life Questionnaire
mPC: Metastatic Prostate Cancer
PC: Prostate Cancer
PCSS: The Prostate Cancer Symptom Scale
QoL: Quality of Life
